# Supplementary material for: The Systems Biology Research Tool: evolvable open-source software
Source: BMC Syst Biol. 2008 Jun 29;2:55. doi: 10.1186/1752-0509-2-55 (PMC2446383; doi:10.1186/1752-0509-2-55)
Supplement: Additional file 1 — SBRT Archive. An archive of the current version of the Systems Biology Research Tool. [file 1752-0509-2-55-S1.zip › sbrt-1.4.0/doc/developers_guide/api/sbrt/kernel/math/geom/ConvexPolytope.html]

ConvexPolytope


|  |  |  |
| --- | --- | --- |
| **Overview** \| **Package** \| **Class** \| **Use** \| **Tree** \| **Deprecated** \| **Index** \| **Help** | |  |
| PREV CLASS   **NEXT CLASS** | **FRAMES**    **NO FRAMES**     **All Classes** |  |
| SUMMARY: NESTED \| FIELD \| CONSTR \| METHOD | DETAIL: FIELD \| CONSTR \| METHOD |  |


---


## sbrt.kernel.math.geom Interface ConvexPolytope<V>

**Type Parameters:**: `V` - the variable type.

**All Known Implementing Classes:**: HashConvexPolytope, HashConvexPolytopeOld

---

``` public interface ConvexPolytope<V> ```

This interface is used to represent convex polytopes that are represented
as the intersection of a finite set of half-spaces.

**Author:**
:   This interface was written and documented by
    Jeremiah Wright while in the Wagner lab.

---

| **Method Summary** | |
| --- | --- |
| `int` | `dimensions()`             Returns the dimensions of the space in which this polytope lies. |
| `java.util.Set<ParallelHyperplanes<V>>` | `getBoundingPlanes()`             Returns the set of bounding hyperplanes that define this convex polytope. |
| `java.util.Set<V>` | `getVariables()`             Returns the variables contained in the hyperplanes defining the bounding half-spaces. |
| `boolean` | `isVariable(java.lang.Object variable)`             Indicates if the provided object is a variable in the bounding hyperplanes of this convex polytope. |
| `boolean` | `isWithin(java.util.Map<? extends V,java.lang.Double> point, double tolerance)`             Indicates if the provided point lies within this convex polytope. |

**Method Detail**

### getVariables

```
java.util.Set<V> getVariables()
```

:   Returns the variables contained in the hyperplanes defining the bounding
    half-spaces.

    :   **Returns:**: the variables contained in the hyperplanes defining the bounding half-spaces.

---


### isVariable

```
boolean isVariable(java.lang.Object variable)
```

:   Indicates if the provided object is a variable in the bounding
    hyperplanes of this convex polytope.

    :   **Parameters:**: `variable` - the potential variable. **Returns:**: `true` if the provided object is a variable in the bounding hyperplanes of this convex polytope; `false` otherwise.

---


### getBoundingPlanes

```
java.util.Set<ParallelHyperplanes<V>> getBoundingPlanes()
```

:   Returns the set of bounding hyperplanes that define this convex polytope.

    :   **Returns:**: the set of bounding hyperplanes that define this convex polytope.

---


### isWithin

```
boolean isWithin(java.util.Map<? extends V,java.lang.Double> point,
                 double tolerance)
```

:   Indicates if the provided point lies within this convex polytope.

    :   **Parameters:**: `point` - variables mapped to numerical values. Every key of this map must be a variable of this polytope and every variable must be present.: `tolerance` - the amount by which a point may lay outside this polytope, but still considered within it. This should be a small positive numer, like 1E-9 for example. **Returns:**: `true` if the provided point lies within this polytope; `false` otherwise.

---


### dimensions

```
int dimensions()
```

:   Returns the dimensions of the space in which this polytope lies.

    :   **Returns:**: the dimensions of the space in which this polytope lies.


---


|  |  |  |
| --- | --- | --- |
| **Overview** \| **Package** \| **Class** \| **Use** \| **Tree** \| **Deprecated** \| **Index** \| **Help** | |  |
| PREV CLASS   **NEXT CLASS** | **FRAMES**    **NO FRAMES**     **All Classes** |  |
| SUMMARY: NESTED \| FIELD \| CONSTR \| METHOD | DETAIL: FIELD \| CONSTR \| METHOD |  |


---
